# Plug for the parasitophorous duct: a solution of two conundra

**DOI:** 10.1186/s12936-020-03445-9

**Published:** 2020-10-16

**Authors:** Prapon Wilairat, Saranya Auparakkitanon

**Affiliations:** 1 Department of Biochemistry, Faculty of Science, Mahidol University, Rama 6 Road, Bangkok, 10400 Thailand; 2 Department of Pathology, Faculty of Medicine Ramathibodi Hospital, Mahidol University, Rama 6 Road, Bangkok, 10400 Thailand

**Keywords:** *Plasmodium falciparum*, Merozoite egress, Merozoite invasion, Parasitophorous duct, Parasitophorous vacuolar membrane, Plug, Red blood cell, Serum protein uptake, Tight junction

## Abstract

**Background:**

We present two conundra in the biology of intraerythrocytic malaria parasite: how an apparent open parasitophorous duct provide direct access of only a select set of serum proteins to the parasitophorous vacuole, and how proteases mediate membrane lysis to allow merozoite egress.

**Solution:**

We posit the existence of a parasitophorous vacuolar duct plug that is originally formed from a tight junction (or parts thereof) between merozoite apical surface and red blood cell plasma membrane, which by moving over the parasite surface towards the posterior end draws the parasite into the host cell interior, and by remaining at the passage orifice provides a location of transporter(s) for import of serum proteins into parasitophorous vacuole and an opening for merozoite egress upon its dissolution/dismantling through protease(s) action.

**Conclusion:**

This notion obviates the need of a distinct intact parasitophorous vacuolar membrane, which in the proposed model is an extension of the red blood cell membrane but still forms an intracellular compartment for parasite growth and development. The model is testable using existing high-resolution electron and X-ray tomography tools.

## Background

Two recent publications in this Journal have recently demonstrated direct access of intraerythrocytic *Plasmodium falciparum* to a selective group of serum proteins via a process independent of passage through infected red blood cell (RBC) cytosol, namely, that of prothrombin, vitamin K-dependent protein S, and vitronectin reported by Tougan et al*.* [1], who identified the presence of such proteins by shotgun liquid chromatography-mass spectrometry/mass spectrometry, western blotting and confocal microscopy of fluorescent-tagged proteins, and that of plasminogen reported by Maluf et al*.* [2] employing immunogold electron microscopy and western blotting. A similar observation and conclusion were earlier made by Tahir et al*.* [3] using biotin- and ^125^I-labelled serum proteins. These findings are reminiscent of the concept of a “parasitophorous duct” proposed by Pouvelle et al. [4] to allow direct access of the outside environment to parasitophorous vacuole (PV) space and into tubules projecting from it, a model that was treated with scepticism based largely on a lack of robustness of the fluorescent probe-labelling technique originally employed to track the route of tagged proteins from the external medium into the parasite. Subsequent detailed examination by transmission electron microscopy of serial sections of fixed and stained mature parasites revealed apparent membrane continuity between red blood cell membrane (RBCM) and parasitophorous vacuolar membrane (PVM) that can leave the parasite exposed to the external medium [5]; in addition, laser scanning confocal microscopy demonstrated carboxylate and amidine-modified fluorescent latex spheres up to 50–70 nm in diameter have direct access to intraerythrocytic parasites, these sizes being consistent with the dimensions of the parasitophorous duct as shown by electron microscopy. However, the most glaring weakness of the existence of a parasitophorous duct is the requirement of an opening at the infected RBC surface of a defined diameter to allow entry of macromolecules into the parasite PV, but without any explanation for the necessary regulation of a consequential intermingling of PV non-macromolecular content with the external milieu, let alone the osmotic pressure arising from an opening at a cell surface.

### Parasitophorous duct plug

**Fig. 1:**
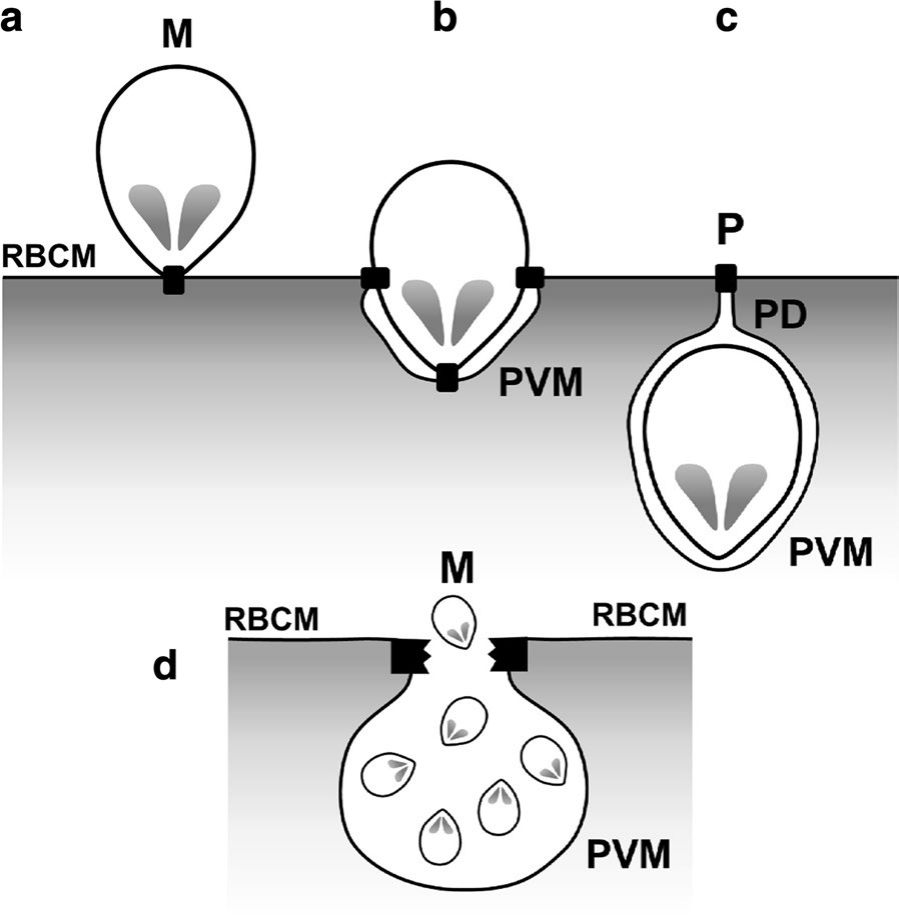
Proposed model for formation of parasitophorous duct plug. **a** Formation of tight junction (black rectangle) between merozoite apical and red blood cell (RBC) membranes. **b** Location of tight junction during entry of merozoite into RBC interior. **c** Parasitophorous duct plug formed from tight junction located at orifice of parasitophorous vacuole upon completion of merozoite ingress. **d** Disruption of parasitophorous duct plug by merozoite protease(s) to form opening on RBC surface for progeny merozoites release. M, merozoite; P, parasitophorous duct plug; PD, parasitophorous duct; PVM, parasitophorous vacuolar membrane; RBCM, red blood cell membrane

In order to solve this apparent conundrum, we propose a parasitophorous duct that is sealed by a “plug” composed of a protein complex, impermeable to all solutes but containing transporters for entry of a select set of serum proteins, i.e. a modified tight junction. This plug originates from the mechanism of merozoite ingress into RBC, a process initiated by formation of an attachment complex between the apical end of an invading merozoite and host cell surface, first reported through painstaking analysis of transmission electron micrographs by Aikawa et al*.* [6], followed by movement of this attachment complex (in the form of a belt-like tight junction) over the merozoite surface towards the posterior end, driven by an actin-myosin motor system located beneath the merozoite plasma membrane and connected to the tight junction endodomains [7]. When the tight junction complex converges at the merozoite posterior region, it is believed that the tight junction complex disassembles allowing the apposed RBC plasma membranes to fuse and generate two separate intact membranes, namely, PVM and RBCM. Applying Occam’s razor, we posit that the tight junction positioned at the RBC surface remains intact, interacting with the two apposed RBCMs, thus forming the plug, but allows detachment of the merozoite to reside and develop within the PV (Fig. 1). The tight junction transmembrane domains would prevent any lateral diffusion of integral membrane proteins across this barrier, consistent of the presence of distinct sets of PVM and RBCM proteins [8, 9]. This model allows normal lateral movement of lipids as expected of a continuous bilayer. Once an external protein gains entry to the PV, its distribution within the tubovesicular network (TVN) and Maurer’s clefts (formed by budding from TVN [10]) is assured [2, 5]. If a protein transporter exists in the parasitophorous tight junction pore structure, might not a similar transporter exist on the merozoite plasma membrane, thereby affording an explanation for the appearance of serum protein(s) in parasite cytosol [1, 2, 5]? As regards the selectivity and specificity of the putative protein transporter(s), it is worth noting the internalized serum proteins vitamin K-dependent protein S and prothrombin have a γ-carboxyglutamic acid-rich domain and vitronectin that does not contain this domain forms a complex with thrombin in serum [11], and plasminogen is found on infected RBC surface and could be a bystander in the uptake process as its presence in internalized parasite is 40- and 100-folds less than that of prothrombin and vitamin K-dependent protein S respectively [2].

The presence of an intact discrete PVM poses the second conundrum: requirement of PVM lysis followed by that of infected RBCM for merozoite egress, an event requiring activation of a cascade of parasite proteases [12], but the exact mechanism of dissolution of the two lipid bilayers by protease(s) remains unclear. This conundrum is readily solved by the existence of the protein plug, degradation/destabilization of which causes restoration of the pore opening that enlarges through lateral sheer stress exerted by the elastic infected RBCM [13], thereby raising the PVM to RBC surface and expelling the progeny merozoites (probably also aided by osmotic pressure) through an apparent opening and in a fixed trajectory. This phenomenon of merozoites’ egress has been directly visualized using high-speed differential interference contrast video microscopy and epifluorescence [14]. Subsequent shearing of the PVM fabric would account for the infected RBCM contortions observed at the edge of the egress opening [15]. The model does not exclude the distinct possibility that the porin-containing PVM [16] is ruptured (by osmotic pressure) *during but not before* merozoites’ egress through the opening in RBCM, and given the rapidity of the egress events (a matter of seconds [16, 17]) it *may appear* that PVM rupture occurs prior to merozoite expulsion.

Is there any evidence for the existence of this conjectural parasitophorous duct plug? Baum et al*.* [18] have recounted the steps leading to obtaining a high-resolution 3-dimensional image of a merozoite invading RBC, which depicts the progression starting with a small apical tight junction that then moves to encircle the merozoite and finally seals the entry at the posterior end. It remains to be shown if this tight junction plug remains at infected RBCM blocking the entrance of the parasitophorous pore. We believe this is a testable model though application of such imaging tools as cryo-X-ray tomography and super-resolution fluorescence microscopy, which already have been applied to the invasion process [19].

## Conclusions

Demonstration of the existence of a plug with protein transporting properties at the entrance to malaria parasite parasitophorous vacuole duct and its dissolution to allow merozoite egress will open up discoveries of new druggable targets impacting intraerythrocytic parasite growth, development and transmission. The magic word (proteases) to unlock Alibaba’s cavern is known; the quest now is to locate the entrance and let the Phoenix rise.

## Data Availability

Not applicable.
